# Falling From Laughing: Laughing Gas-Induced Subacute Combined Degeneration From Nitrous Oxide

**DOI:** 10.7759/cureus.62138

**Published:** 2024-06-11

**Authors:** Abhinav K Rao, Fahim Syed, Thomas J Lee, Gilberto U Umanzor, Jeffrey Bodle

**Affiliations:** 1 Internal Medicine, Trident Medical Center, Charleston, USA; 2 Internal Medicine, Rutgers University New Jersey Medical School, Newark, USA; 3 Neurology, Trident Medical Center, Charleston, USA

**Keywords:** nitrous oxide, neurology, subacute combined degeneration, nitrous oxide abuse, laughing gas

## Abstract

Laughing gas is becoming increasingly popular as a recreational drug of choice, particularly among young adults. Nitrous oxide, the toxic component of laughing gas, can cause neuronal injury when used in high doses. Through multiple mechanisms, nitrous oxide leads to B12 depletion and subsequent demyelination, particularly in the spinal cord. Here, we present the case of a 27-year-old female who presented with ataxia and was found to have laughing gas-induced subacute combined degeneration from nitrous oxide. After aggressive vitamin B12 repletion and laughing gas cessation for three months, the patient improved.

## Introduction

Nitrous oxide (N_2_O), commonly known as laughing gas, has gained popularity as a recreational drug in recent years, particularly among adults under 30 years of age [1]. Traditionally used for analgesia and sedation, N_2_O induces rapid sedation, euphoria, and, occasionally, hallucinogenic effects. The effects are short-lived, leading to repeated inhalations from a balloon to increase cumulative drug exposure. Some studies have reported up to 10 balloons of N_2_O used per episode [2-5]. High levels of N_2_O can lead to toxicity through various mechanisms, with vitamin B12 depletion emerging as a key factor [5]. Here, we present the case of a 27-year-old female who presented with gait ataxia and abnormal sensation found to have laughing gas-induced subacute combined degeneration from nitrous oxide. Clinicians should be aware of this emerging recreational drug as an important cause of new-onset neurological impairment.

## Case presentation

A 27-year-old female with a past medical history of anxiety, depression, and opioid use disorder was brought by Emergency Medical Services with an inability to stand, lower extremity paresthesias, and gait instability. Starting two months ago, she stated that her legs would intermittently “fall asleep.” One month later, she developed paresthesia in both lower extremities. She reported associated fatigue and brain fog.

In the week before admission, her paresthesia worsened to the point where she could not walk without support. On the day of admission, her symptoms progressed to a complete loss of sensation in her legs and an inability to stand, prompting her to seek medical attention.

Her social history revealed daily consumption of about five alcoholic drinks and regular inhalation of N_2_O canisters. She initially began recreational inhalation of N_2_O six months before admission and reported a gradual increase in the quantity inhaled. Around the time her neurological symptoms began (two months before admission), she inhaled approximately 320 g of N_2_O per month (40 N_2_O canisters in the month). In the days leading up to her admission, her N_2_O inhalation had increased significantly, reaching around 2,560 g over six hours each day (approximately 320 N_2_O canisters over six hours each day).

On physical examination, she appeared in no acute distress. Oropharynx examination revealed a darkened, smooth red tongue. Neurological evaluation of the dorsal columns revealed a positive Romberg sign and ataxic gait. Additionally, sensory deficits included symmetric paresthesia distally from the umbilicus, reduced light touch and pinprick sensation throughout both upper and lower extremities without a clear sensory level, and bilateral pronator drift. Finger-to-nose testing revealed a serpentine pattern. Reflexes were 1+ and symmetric throughout. Cranial nerves II-XII were intact, and the Babinski sign was absent. Motor strength was 5/5 in all extremities.

On admission, laboratory data revealed a low vitamin B12 level (78 pg/mL, reference range: 180-914 pg/mL), high methylmalonic acid (MMA) level (4,137 nmol/L, reference range: 0-378 nmol/L), and normocytic anemia (Table 1). The urine drug screen was positive for benzodiazepines but negative for opioids, barbiturates, PCP, amphetamines, cocaine, cannabinoids, and alcohol. Syphilis was not checked as the patient was not sexually active. The patient denied alcohol as well as intravenous and other recreational drug use.

**Table 1:** Laboratory data. H: high values of laboratory data; L: low values of laboratory data

	Values	Reference range
Sodium	138	136–145 mEq/L
Potassium	3.8	3.6–5.1 mEq/L
Chloride	105	101–111 mEQ/L
Carbon dioxide	26	22–32 mEq/L
Anion gap	7	3–13
Glucose	106 (H)	70–100 mg/dL
Creatinine	0.6 (L)	0.7–1.2 mg/dL
Calcium	8.8 (L)	8.9–10.3 mg/dL
Total protein	7.3	6.1–8.0 g/dL
Albumin	3.8	3.4–4.8 g/dL
Total bilirubin	1.1 (H)	<1.0 mg/dL
Aspartate transaminase	21	<35 U/L
Alanine transaminase	22	10–63 U/L
Alkaline phosphatase	50	32–101 U/L
C-reactive protein	5.2 (H)	0–1 mg/dL
Folic acid	16.2	>5.9 ng/mL
Magnesium	2.2	1.8–2.5 mg/dL
Thyroid-stimulating hormone	1.41	0.34–5.60 mU/L
Vitamin B12	78 (L)	180–914 pg/mL
Methylmalonic acid	4,137 (H)	0–378 nmol/L
White blood cell	6.8	4.0–15.7 k/mm^3^
Hemoglobin	10.7 (L)	13.5–16.5 g/dL
Mean corpuscular volume	94	80–95 fL
Platelet	274	135–350 k/mm^3^
A1c	5.0	4.8–6.0%
Erythrocyte sedimentation rate	26 (H)	9–20 mm/hour

**Figure 1:**
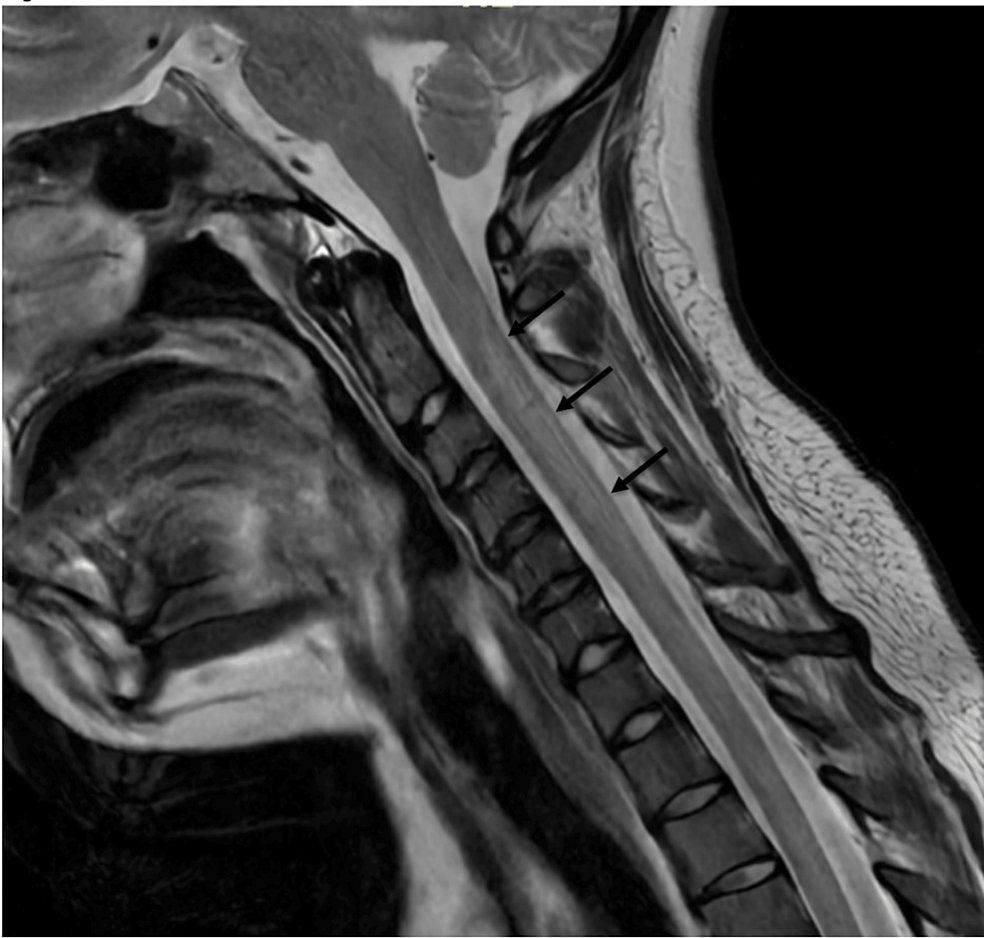
Laughing gas-induced subacute combined generation from nitrous oxide. MRI of the cervical and thoracic spine with and without contrast revealed a T2 hyperintensity (arrows) within the dorsal columns of the spinal cord, most severe throughout the upper cervical spinal cord extending from C2 through C5-C6.

**Figure 2:**
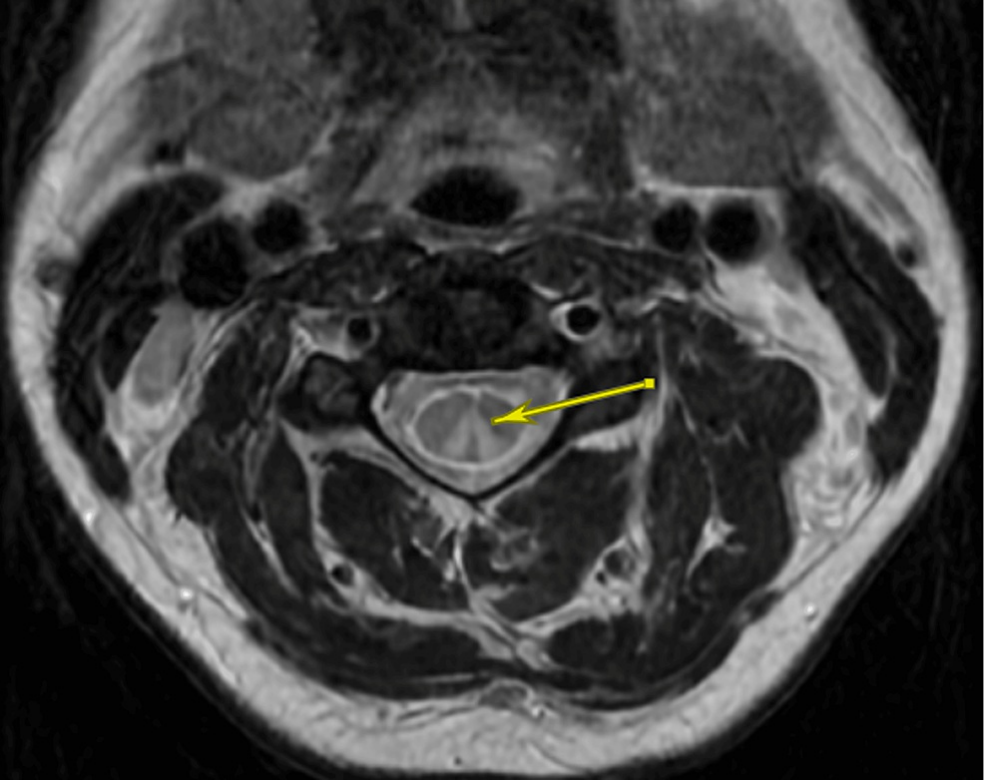
T2 hyperintensity most prominent in the dorsal columns suggestive of subacute combined degeneration. MRI of the cervical spine (Axial, T2 fast spin echo) at the C2-C3 level showing T2 hyperintensity within the dorsal columns.

A non-contrast CT scan of the head showed no acute changes, and an MRI of the brain with and without contrast was also unremarkable. However, further imaging with MRI of the cervical and thoracic spine with and without contrast revealed a signal abnormality within the dorsal columns of the spinal cord, most severe throughout the upper cervical spinal cord extending from C2 through C5-C6 (Figures 1, 2). These findings were highly suspicious for subacute combined degeneration.

The patient began daily intramuscular vitamin B12 replacement with 1,000 µg weekly and was discharged from the hospital for physical rehabilitation and close neurology follow-up as an outpatient. Within three months of discontinuing N_2_O inhalation and receiving vitamin B12 supplementation, the patient experienced significant improvement in sensory and motor function, including regaining the ability to walk.

## Discussion

N_2_O, or laughing gas, has gained recognition as a recreational drug in recent years, particularly among 16- to 24-year-olds, where it ranks as the second most-used drug behind cannabis in the United Kingdom [1]. High levels of N_2_O lead to toxicity through multiple mechanisms, with vitamin B12 depletion as a driving factor. Patients at risk for vitamin B12 deficiency have a lower threshold for N_2_O-induced toxicity [2].

Vitamin B12 is inactivated in N_2_O toxicity through the irreversible binding of N_2_O to the cobalt ion in vitamin B12 [3]. Normally, vitamin B12 is a cofactor for methionine synthase and methylmalonyl-CoA mutase. Dysfunction of these enzymes due to vitamin B12 inactivation leads to neurological and hematological abnormalities through malfunctioning catabolism of protein and fatty acids.

In fatty acid oxidation, methylmalonyl-CoA is converted to succinyl-CoA by methylmalonyl-CoA mutase, which uses vitamin B12 as a cofactor. When vitamin B12 is depleted, methylmalonyl-CoA mutase becomes dysfunctional. This leads to decreased succinyl-CoA synthesis and increased MMA, the precursor to methylmalonyl-CoA. Excess MMA leads to neurodegeneration by impairing spinal cord myelinization. Proposed mechanisms include inhibition of complex II, which is involved in the tricarboxylic acid (TCA) cycle and mitochondrial electron transport chain, decreased TCA activity due to reduced succinyl-CoA substrate, and synergistic excitotoxicity [4]. A recent study showed that patients with N_2_O overdose exhibited more prominent axonal dysfunction than those with vitamin B12 deficiency alone, suggesting another mechanism contributing to N_2_O-induced neurotoxicity [5].

In addition to neurological dysfunction, vitamin B12 deficiency leads to hematologic abnormalities through the dysfunction of methionine synthase. This enzyme uses methyl tetrahydrofolate (THF) and vitamin B12 as cofactors to convert homocysteine to methionine. During this process, methyl-THF is converted back to THF, which is a substrate required for DNA synthesis. If THF cannot be regenerated, as in vitamin B12 deficiency, DNA synthesis will be impaired. This results in ineffective erythropoiesis, characterized by defective DNA synthesis leading to megaloblastic transformation and intramedullary hemolysis [5]. Although erythroid hyperplasia occurs, the erythroid cells do not mature normally and subsequently die in the bone marrow. This can lead to a combination of megaloblastic anemia and cytopenias. Additionally, elevated homocysteine levels increase cardiovascular risk. Hyperhomocysteinuria elevates the risk of thrombosis and atherosclerosis, potentially causing stroke and myocardial infarction [6].

A thorough history is critical in making a diagnosis. Clinical features include hematological, neuropsychiatric, thrombotic, and cutaneous presentations of N_2_O overdose [7]. N_2_O has also been hypothesized to inhibit responses to hypoxia and hypercapnia, which has resulted in mortality [8]. The analgesic effect of N_2_O involves several neuromodulators in the spinal cord and resembles that of opioids. Moreover, N_2_O’s anxiolytic effect may be due to its similarity to benzodiazepines, potentially binding to select subunits of the GABA (A) receptors. This may also contribute to its anesthetic effect, with possible involvement of the NMDA receptor [9].

Laboratory tests may reveal features suggestive of vitamin B12 deficiency. A vitamin B12 level is easy to check and should be measured. If there is uncertainty, measuring MMA may help, as elevated levels are exclusive to B12 deficiency compared to other nutritional deficiencies such as folate deficiency [4]. Features of intramedullary hemolysis, such as elevated lactate dehydrogenase, low haptoglobin, and indirect hyperbilirubinemia, may also be observed [9]. A complete blood count may show macrocytic anemia due to megaloblastic transformation.

Radiographic findings may include signal abnormalities within the dorsal columns of the spinal cord, particularly in the cervical and thoracic regions [3]. Demyelination in the spinal cerebellar tracts, corticospinal tracts, and dorsal columns leads to the host of symptoms seen in subacute combined degeneration. Patients will present with ataxia and paresthesia as seen in this patient, as well as impaired proprioception and altered sensorium. Dementia can be seen in the elderly population presenting with subacute combined degeneration [5].

Treatment involves repleting vitamin B12, which is typically accomplished with B12 injections, and N_2_O cessation. Monitoring for B12 injection-induced hypokalemia during the first 48 hours of treatment is important, as new red blood cell production after providing the necessary materials for DNA synthesis may lead to potassium uptake into cells [2]. Aggressive vitamin B12 repletion and drug abstinence can reverse neurological impairment and improve functional outcomes, as seen in this case.

## Conclusions

We present a rare case of subacute combined degeneration induced by N_2_O overdose. Clinicians should consider this emerging recreational drug-related cause of new neurological deficits in their differential diagnosis. Patients should be counseled on the importance of discontinuing N_2_O use and promptly starting vitamin B12 repletion to reverse neurological symptoms.

## References

[1] van Amsterdam J, Nabben T, van den Brink W (2015). Recreational nitrous oxide use: prevalence and risks. Regul Toxicol Pharmacol.

[2] Buhre W, Disma N, Hendrickx J (2019). European Society of Anaesthesiology Task Force on Nitrous Oxide: a narrative review of its role in clinical practice. Br J Anaesth.

[3] Edigin E, Ajiboye O, Nathani A (2019). Nitrous oxide-induced B12 deficiency presenting with myeloneuropathy. Cureus.

[4] Okun JG, Hörster F, Farkas LM (2002). Neurodegeneration in methylmalonic aciduria involves inhibition of complex II and the tricarboxylic acid cycle, and synergistically acting excitotoxicity. J Biol Chem.

[5] Tani J, Weng HY, Chen HJ, Chang TS, Sung JY, Lin CS (2019). Elucidating unique axonal dysfunction between nitrous oxide abuse and vitamin B12 deficiency. Front Neurol.

[6] Shibeeb S, Abdallah A, Shi Z (2024). Blood homocysteine levels mediate the association between blood lead levels and cardiovascular mortality. Cardiovasc Toxicol.

[7] Oussalah A, Julien M, Levy J (2019). Global burden related to nitrous oxide exposure in medical and recreational settings: a systematic review and individual patient data meta-analysis. J Clin Med.

[8] Bäckström B, Johansson B, Eriksson A (2015). Death from nitrous oxide. J Forensic Sci.

[9] Emmanouil DE, Quock RM (2007). Advances in understanding the actions of nitrous oxide. Anesth Prog.

